# Social Cognition Deficits: Current Position and Future Directions for Neuropsychological Interventions in Cerebrovascular Disease

**DOI:** 10.1155/2017/2627487

**Published:** 2017-07-03

**Authors:** Progress Njomboro

**Affiliations:** Department of Psychology, PD Hahn, University of Cape Town, Cape Town, South Africa

## Abstract

Neuropsychological assessments of cognitive dysfunction in cerebrovascular illness commonly target basic cognitive functions involving aspects of memory, attention, language, praxis, and number processing. Here, I highlight the clinical importance of often-neglected social cognition functions. These functions recruit a widely distributed neural network, making them vulnerable in most cerebrovascular diseases. Sociocognitive deficits underlie most of the problematic social conduct observed in patients and are associated with more negative clinical outcomes (compared to nonsocial cognitive deficits). In clinical settings, social cognition deficits are normally gleaned from collateral information from caregivers or from indirect inferences made from patients' performance on standard nonsocial cognitive tests. Information from these sources is however inadequate. I discuss key social cognition functions, focusing initially on deficits in emotion perception and theory of mind, two areas that have gained sizeable attention in neuroscientific research, and then extend the discussion into relatively new, less covered but crucial functions involving empathic behaviour, social awareness, social judgements, and social decision making. These functions are frequently impaired following neurological change. At present, a wide range of psychometrically robust social cognition tests is available, and this review also makes the case for their inclusion in neuropsychological assessments.

## 1. Introduction

Cerebrovascular disease, including conditions related to large infarcts, multiple microinfarcts, lacunes, subcortical arteriosclerotic leukoencephalopathy, and haemorrhages, contributes a significant healthcare burden to societies and is one of the leading causes of disability worldwide [1, 2]. Cerebrovascular disease is also common in patients with degenerative disorders, suggesting that a causative role is some of them. For instance, concurrent cerebrovascular disease is a common neuropathological finding in dementing illness, particularly in Alzheimer's disease [3], where its comorbidity is also associated with relatively worse cognitive deficits and a lowering of the threshold for dementia [4, 5].

Studies and reviews investigating neurocognitive profiles associated with cerebrovascular disease predominantly focus on basic cognitive functions, such as episodic, semantic, and working memory; perceptual speed, attention, and visuospatial abilities, for example, [6–10]. Whereas focal lesions resulting from cerebrovascular disease may cause distinct sociocognitive deficits (such as the impaired recognition of some emotions following amygdala, ventromedial prefrontal cortex, or insular lesions), the neurological changes associated with more diffuse damage disrupt frontal-subcortical pathways and fronto-temporal connections that make up the “social brain”; a distributed brain network responsible for social cognition functions crucial for adaptive social behaviour [11, 12]. Social cognition impairment is therefore common sequelae of cerebrovascular illness [13] and often leads to disordered interpersonal functioning and poor regulation of personal conduct.

Although the behavioural dysfunction that follows cerebrovascular disease is sometimes seen in the context of deficits in the basic cognitive functions mentioned above, most of it has its basis in dysfunctional cognitive abilities that serve a more social function. These sociocognitive functions play an important role in adaptive social behaviour by enabling the perception and interpretation of other people's intentions, dispositions, and behaviours during social exchange and are also crucial in the generation of contextually relevant and appropriate social responses [14]. Consequently, due to damage in areas of the brain that subserves these functions, a significant number of patients with cerebrovascular disease often show conduct that violate social norms and conventions. They may also become socially withdrawn, disengaged, and isolated [15]. Such social isolation is associated with faster cognitive decline and higher mortality [16]. Furthermore, impaired sociocognitive processing makes patients vulnerable to social exploitation and scams [17].

Clinically, social cognition deficits are associated with poorer functional outcomes and are better predictors of postinjury recovery compared to nonsocial cognitive deficits [18]. They are associated with a much heavier caregiver burden [19, 20] and much higher familial stress [21–27]. Caregivers often report a higher frequency of problematic changes in personality in patients, such as becoming more self-centred, more disliking of others, and more quarrelsome [28]. As a result, sociocognitive impairment has significant negative effects on patients' social competence and ability to thrive and function in the community and tends to shrink patients' social opportunities over time [29]. Even though research highlight the clinical importance of social cognition deficits and confirm the utility of targeted interventions against them, they remain underdiagnosed and undertreated.

Here, I make the case for the inclusion of social cognition measures in the neuropsychological assessment of people who suffer from cerebrovascular disease and also highlight some of the important issues that need to be considered in incorporating such measures into these assessments. In clinical settings, sociocognitive dysfunction is often gleaned indirectly from collateral information given by patients' caregivers or significant others, as well as from inferences made from results of patients' performance on standard cognitive tests. Information from these indirect sources is valuable but does not identify the finer aspects of the social cognition deficits or quantify them in a manner that enables intervention.

## 2. Conceptual Overview of Social Cognition

Social cognition refers to a wide range of cognitive capacities that enable humans to understand themselves, communicate with and understand others, store and use information about culturally acceptable norms and behavioural scripts, and engage in appropriate goal-directed behaviour [30–32]. These capacities include the ability to decode and understand social cues like emotional expressions on a face, in the voice, or from body posture and also include higher order functions that rely on these cues for making inferences about other people's mental states (e.g., theory of mind reasoning), making moral decisions, regulating emotions and feelings, and experiencing and expressing empathy [33, 34].

Conceptually, it is not very clear whether functions that fall under the broad domain of social cognition are markedly distinct from one another and empirical evidence for those distinctions is inconclusive. For instance, while many would treat emotion perception and theory of mind (ToM) as distinct processes, emotion perception can be seen as a subcomponent of ToM processing [35]. Emotion perception can also be viewed as a crucial substep in empathic processing, since one needs to correctly recognise someone's emotional state first, before other higher order processes operate on the perceived emotion to elicit empathic reactions. Data from lesion and brain imaging studies however suggest that the brain networks that play critical roles in some of the social cognition domains are largely distinct, although they may also overlap significantly [11, 36]. In some instances, a distinction can also be made between “hot” social cognition involving emotion processing and empathic responding, versus “cold” social cognition related mainly to understanding other people's mental states.

Adolphs [37] offers a more integrated model of social cognition processes. Broadly, he groups these processes into three categories; the first category is comprised of perceptual or representational sociocognitive elements (e.g., those involved in the perception of face and speech information, biological motion, and the representation of socially relevant movements). The second category is made up of evaluative or interpretational processes (e.g., those involving emotion recognition, emotional and cognitive empathy, theory of mind, and the use of pragmatics), and the third category is made up of regulatory processes (e.g., processes for emotion regulation, self-awareness and reflection, cognitive control, and reappraisal). See Kennedy and Adolphs [38] for a more involved discussion on this.

A study by Pinkham et al. [39] identifies emotion processing, social perception, theory of mind/mental state attribution, and attributional style/bias as the key domains that make up social cognition. In their factor analysis of social cognition studies, they found a distinction of social cognition domains based on the level of information processing involved (i.e., perception versus inferential versus regulatory processing) rather than the one based on categories of social information like “emotions” or “mental states” [39]. A workshop on social cognition in schizophrenia also identified ToM, social perception, social knowledge, attributional bias, and emotional processing as the main social cognition domains relevant to that disorder [14], although it would be important to investigate whether these same domains would be equally relevant in relation to social cognition following cerebrovascular disease. What these studies indicate is that while previous research has mainly focused on sociocognitive processes involved in ToM reasoning and emotion recognition, social cognition encompasses a range of processes that go beyond these two.

## 3. Relationship between Social and Nonsocial Cognition

One point of view suggests that social cognition evolved as a result of the specific need for humans to survive in social groups, thereby implying that social cognition is distinct and independent of nonsocial information processing capacities [12, 37]. Fiske [40] also points out that the distinctive feature in social cognition is its primary involvement in “thinking about people.” In this context, social cognition is viewed as a component of cognitive performance, and what distinguishes it from non-social cognition are the contents of representations on which general cognitive processes are made to operate. The implication from this position is that there would be some degrees of overlap between social and nonsocial cognition [38, 41].

Quite often, sociocognitive problems occur in patients who have relatively intact nonsocial neurocognitive functions. A number of models also report dissociations between social cognition deficits and performance on standard (nonsocial) neurocognitive tests, suggesting that some brain networks may be specifically dedicated to social cognition [38, 42–44]. The classic case of Phineas Gage illustrates this dissociation quite well. Although Gage exhibited dysfunctional social conduct following his brain damage, his intellectual capacities, as well as other basic cognitive processes like language and attention, remained largely intact [45]. Jacobs et al. [46] also found that patients with Parkinson's disease have impaired social cognition in relation to emotional facial imagery, in the presence of intact imagery for objects. For more examples of these social/nonsocial cognition dissociations, see also [47–49]. However, see Uhlhaas et al. [50].

There is little doubt though that nonsocial neurocognitive processes are crucial in directing and controlling some aspects of social cognition. For instance, capacities like processing speed, cognitive flexibility, preinjury intellectual functioning, and working memory affect performance on sociocognitive tasks [28, 34, 51].

## 4. Social Cognition Domains

### 4.1. Emotion Recognition

Emotion recognition involves perceptual capacities for decoding and making meaning out of emotional expressions. Following neurological change, the ability to decode emotions from facial expressions can be independently impaired in the presence of intact face processing [34, 52, 53]. Impaired recognition of emotion is usually worse for vocally expressed emotions [28, 34, 52], and longitudinal studies find that emotion recognition deficits following brain damage are long term and enduring [34, 53]. For instance, a meta-analysis by Babbage et al. [54] showed that up to 39% of people with moderate to severe brain injury suffer long-term significant deficits in recognising emotions from facial expressions. Similar deficits are also seen in survivors of stroke [55]. See also Bornhofen and Mcdonald [29] and Radice-Neumann et al. [56].

Brain imaging studies implicate a wide neural network involving frontotemporoparietal areas and parts of the limbic system in the processing of facial expressions of emotion [12]. Some of these areas are relatively more vulnerable to cerebrovascular disease, which explains the high prevalence of emotion recognition deficits in patients [56, 57]. Genova et al. [57] suggest that a pattern of white matter and grey matter damage underlies impaired affect recognition. Their study incorporated structural neuroimaging including diffusion tensor imaging techniques to investigate white matter integrity and its relation to facial affect recognition. They found significant associations between reduced white matter integrity in the inferior longitudinal fasciculus and inferior-fronto-occipital fasciculus and reduced facial affect recognition. Poor emotion recognition is also associated with reduced grey matter volume in the lingual and parahippocampal gyrus.

Patients with cerebrovascular disease are also significantly worse at recognising negative emotions like anger, disgust, sadness, and fear than at recognising positive emotions like happiness, joy, and surprise [54, 58]. This may reflect the relative vulnerability of areas of the brain that process negative emotions (e.g., ventromedial frontal regions, amygdala, and insula) to cerebrovascular disease [12, 59].

Impaired emotion recognition in patients with cerebrovascular disease is often associated with significant communication difficulties, socially inappropriate behaviour, and impoverished social relations [56, 60]. For instance, Pettersen [60] found that participants with acquired brain damage who were poor at recognising emotional facial expressions were also rated by their relatives as showing less socially appropriate behaviour. In another study, Knox and Douglas [51] found that emotion recognition deficits in patients were related to problems in social functioning measured on the Revised Craig Handicap Assessment and Reporting Technique [61].

Tests for emotion recognition mainly target basic/primary emotions (i.e., anger, disgust, fear, sadness, surprise, and happiness) tested on expressory modalities like body posture, the face, and the voice. Much broader approaches evaluate emotional functioning by assessing capacities involved in identifying, facilitating, understanding, and managing emotions but require lengthy administration time frames that may be difficult to fit in with other clinical assessments (e.g., Mayer et al. [62, 63]). These tests may be useful in cases where affective deficits are the dominant clinical presentation.

In clinical settings, assessment for emotion recognition deficits could be restricted to the range of emotions that are commonly associated with significant functional deficits in patients, such as negative emotions. It is also important to control for the presence of affective disorders. Affective disorders are prevalent in cerebrovascular disease [64, 65] and have an influence on emotion perception ability [66, 67]. However, some evidence suggests that the emotion recognition deficits seen in most patients with cerebrovascular illness are due to the injury itself, for example, [34, 52]. See Table 1 for some emotion recognition tests.

### 4.2. Theory of Mind

Theory of mind (ToM) refers to a set of sociocognitive capacities that enable us to decode social cues and use them to infer or reflect on the contents of other people's (and our own) mental state [68]. Impaired ToM processing accounts for significant sociobehavioural impairment following cerebrovascular disease [52, 69, 70]. A meta-analysis of 26 studies by Martín-Rodríguez and León-Carrión [68] whose objective was to compare patients with acquired brain damage to healthy controls on performance on a range of ToM tasks showed moderate to severe ToM impairment in the clinical sample. As mentioned earlier, the high prevalence of ToM deficits in patients with cerebrovascular disease is probably testimony to the vulnerability of the wide neural network that makes up the social brain. As far as ToM is concerned, this network includes the temporoparietal junction [71–73], the medial prefrontal cortex [11], and the inferior dorsolateral and orbitofrontal areas [74].

ToM tests come in all shapes and form—from belief reasoning story tasks to tests that require the inference of mental states from pictures of body postures or pictures of faces or eyes. When assessing ToM in patients with cerebrovascular disease, especially using belief reasoning tasks, it is also important to control for executive function deficits. Patients with frontal damage may perform poorly on belief reasoning tasks not because they have a ToM deficit per se, but because they have executive function deficits that make it harder for them to inhibit their own perspective. Inhibiting one's perspective while attending to the protagonist's perspective is a crucial executive control function in solving false belief tasks [75]. In the same context, patients with global face processing deficits may also perform poorly on those ToM tests that require the decoding of mental or emotional states from face features (e.g., the Eyes in the Mind test).

### 4.3. Empathy

Impaired empathic behaviour is another common disorder following cerebrovascular disease [76–79] and is associated with problematic personality changes such as egocentricity, self-centredness, oppositional behaviour, social disinhibition, aggression, insensitivity to the needs of others, and lack of prosociality [80, 81]. Attenuated empathic behaviour is also frequently associated with significant caregiver distress [19–21, 26].

Current neuroscientific models subdivide processes involved in empathy into mainly cognitive and affective subdimensions. Some researchers add a motoric dimension too, in reference to empathic processes involved in the mirroring of the movements, emotional expressions, and posture of observed others (e.g., Blair [82]). Cognitive empathy relates to capacities that enable us to infer another person's perspective and mental state (some authors do not distinguish between ToM and cognitive empathy). Affective empathy involves capacities that allow for the sharing of other people's emotional states and experiences and also those involved in generating appropriate emotional responses to them [83]. Patients with acquired brain damage are more likely to show deficits in both cognitive and affective empathy [77–79], but deficits in cognitive empathy are associated with higher levels of caregiver distress [84].

Empathic processing recruits a wide network of the social brain, because it involves other aspects of social cognition. For instance, empathy relies on the ability to recognise the emotions and mental states of others and then infer meaning from these based on the environmental context, social knowledge, and personal experience and then generate socially appropriate emotional responses, thoughts, and behaviour. These capacities recruit diverse parts of the social brain including medial prefrontal areas, regions of the temporoparietal junction and temporal poles, and areas that make up the mirror neuron system, as well as emotion pathways and areas in the limbic system [85–88].

Most empathy measures rely on self-report questionnaires to assess empathic experience and behaviour. Due to a lack of solid consensus on the conceptualisation of empathy, the tests also vary in the aspects of empathy that they sample. Some assess perspective taking, while others tap capacities for sympathy, emotional contagion, or one's emotional distress in response to others' predicament, among other related conceptualisations.

### 4.4. Social Judgements and Social Awareness

Cerebrovascular disease can result in deficits in social awareness and problems in making social judgements. For instance, in a study by McDonald and Flanagan [89], patients who had suffered neurological changes could interpret the meaning of comments that were meant to be taken literally but were significantly impaired when required to infer the meaning of interpersonal exchanges between people that encompassed nonliteral sarcastic remarks.

In our own research, we found that patients with an acquired brain damage were impaired in making judgements about the appropriateness of behaviour in social interactions portrayed in story vignettes and also performed abnormally on tasks requiring them to identify social roles or match behaviour to social contexts [90]. Such social judgement deficits may contribute to the inappropriate misconduct frequently seen in patients following brain damage. These socioperceptive dysfunctions associate with lower levels of community functioning and contribute to social isolation [14].

Studies also frequently report impaired social judgements related to moral decision making in patients with brain injury [91]. These moral reasoning deficits are closely linked to problems with processing social emotions like guilt and shame and are thought to reflect attenuated affective influences on goal-directed social behaviour and thought. Tests show that the moral judgement deficits are dissociable from errors due to deficits in declarative knowledge about social and moral norms [92].

Typically, commonly used moral reasoning tests ask participants to respond to hypothetical moral dilemmas (e.g., the trolley problems) that require participants to judge the permissibility of intentionally harming one individual in order to save an aggregate more. Patients with damage to the medial prefrontal cortex tend to judge intentionally harming one person in order to save many more (as in pushing a bulky man in front of a runaway trolley in order to stop it from hitting and killing five others) as morally permissible. Neurologically intact participants on the other hand tend to judge such utilitarian means-to-an-end intentional harm as less permissible [93, 94]. It is thought that moral reasoning on these tasks is initiated and directed by automatic subconscious emotional signals generated through the influence of the medial prefrontal cortex prebiasing social cognition towards more adaptive sociobehavioural choices [92]. Cerebrovascular disease can weaken this mechanism resulting in patients making moral decisions that are predominantly cognitive, intentional and conscious, and therefore utilitarian and socially maladaptive or inappropriate [95].

## 5. Assessment and Screening of Social Cognition Deficits

Direct social cognition tests with good psychometric properties are now available, although care should be taken to ensure that tests are relevant for a particular clinical group. Some of the measures have been developed for use on specific disorders or age groups and frequently produce ceiling effects when used on other clinical populations. This is particularly the case with some ToM tasks [14]. Table 1 lists tests that have been used by researchers to assess some common social cognition functions. These tests may offer an important starting point in creating tests for the neuropsychological screening of social cognition deficits.

Ideally, assessment protocols for sociocognitive deficits should adopt the same format as those for nonsocial neurocognitive deficits. Initially, social cognition dysfunction can be gleaned from a patient's history of present illness and presentation, as well as collateral information from caregivers. This can be followed by a broad but shallow battery of social cognition tests. The emerging clinical picture can be further investigated through more targeted tests. In the same vein, interpretation of test results should be informed by a patient's medical, psychiatric, and neurocognitive profiles, to rule out the influence of other (nonsociocognitive) variables on test performance.

Performance on social cognition tests may be less informative when patients have deficits in ancillary cognitive processes that are not related to sociocognitive functioning but are needed to perform on the tests. For instance, language comprehension deficits can interfere with performance on story-based ToM tasks. Social cognition tasks that require comprehension of complex language may therefore not be suitable. In many instances, victims of cerebrovascular disease have executive function deficits and sociocognitive tasks that require more cognitive control may not be suitable for assessing social cognition in these patients. Administering social cognition tests alongside standard tests for neurocognitive functioning can then help isolate the influence of these nonsocial cognitive processes on performance.

It is also important to note that in some instances, particularly with damage involving the frontal cortex, it is common to find patients with a know-do dissociation. While such patients can perform at normal or near normal levels on offline lab-based socio-cognitive tests, they exhibit significant social cognition impairment in real life. Developing tests sensitive enough to tap into real-life sociocognitive processing is a challenge for researchers. It is therefore imperative that collateral information about the patient's social conduct is also obtained from caregivers and significant others and used alongside results from the social cognition tests.

## 6. Treatment and Rehabilitation of Social Cognition Deficits

Patients' social skills can be improved through targeted individual or group training on isolated aspects of social cognition [96]. Studies using psychosocial interventions targeting specific sociointeractive behaviours like turn taking and making eye contact during conversation have reported some success in some neurologic and psychiatric samples, particularly in improving patients' ability to recognise expressions of emotion. See review by Driscoll et al. [97].

Studies have also reported improvements in social conduct after interventions targeting broader aspects of social cognition. For example, Helffenstein and Wechsler [98] used a 20-hour interpersonal process recall (IPR) training protocol in which a recording of patients' interpersonal interactions was followed by a review of the interactions with feedback being given to patients. Patients who received IPR training had better interpersonal skills compared to those who did not receive the training after a one-month follow-up period. In another study, Bornhofen and Mcdonald [29] administered an 8-week social skills training protocol to 12 patients with acquired brain injury to improve emotion perception. The treatment group significantly improved on the ability to recognise emotions from dynamic facial expressions and also during everyday interactions. Guercio et al. [99] also used a matching-to-sample technique to train patients with acquired brain damage to recognise basic emotions from static pictures of emotional faces and reported an improvement in emotion recognition in the participants after the training. In another study, low physiological arousal to negative faces normalised when patients were given explicit instructions to attend to the images [100]. See also Radice-Neumann et al. [56] and Dahlberg et al. [101] for similar studies.

Training tools using new technologies like virtual reality environments can offer a unique platform to recreate and present controlled real-life social scenarios through which rehabilitation for social cognition problems can be tailored and administered. The main advantage of these environments is that they can offer interactive social spaces without the social costs or pressures found in real-life interactions. Since these environments create social events that are closer to real-life scenarios, they have the added advantage that treatment effects can be generalised to the real world [102].

Medication can also improve social functioning. For instance, use of the neuropeptide oxytocin can improve aspects of social functioning [103–105]. Hurlemann et al. [105] gave participants intranasal oxytocin and found significant positive effects on empathic deficits. Environmental stimulation, for example, music therapy, can also produce positive results [91].

It is worth noting though that most of the interventions mentioned above have been trialled in patients with TBI, neurodegeneration, or psychiatric illness, whose etiological, clinical, and neuropsychological profiles may differ from those of patients with cerebrovascular lesions.

## 7. Future Directions for Research

Despite the common realisation that social cognition deficits have important clinical and functional correlates for patients, there are some challenges in moving the field forward towards a consistent and empirically based assessment in clinical evaluations. For instance, expert consensus surveys and studies investigating the factor structure of sociocognitive domains are needed to create general agreement on the key domains that constitute social cognition. More studies are needed to establish the psychometric robustness of most of the social cognition measures, particularly in terms of their ecological validity.

There is also a need to develop sociocognitive batteries using tasks that have robust psychometric properties, and one way to go about this is to do more validation and standardization studies on the current commonly used tests. The utility of these tests can also be evaluated in terms of their suitability for specific patient populations and of their sensitivity to real-life social functioning deficits. In many instances, simple, easy to administer tests with good control on cognitive load (e.g., with less semantic and executive function loading) would be preferable for most clinical groups.

More recent brain imaging methodologies can also be used to validate social cognition tests. Brain imaging data can be correlated to data on sociocognitive tests and improve our understanding of sociocognitive functions subserved by the network of structures that make up the social brain [11, 12, 36, 87]. Techniques like diffusion tensor imaging (DTI) allow in vivo studies of the anatomy and integrity of white matter tracts in the damaged brain and can help explain relationships between changes in white matter integrity and deficits shown on sociocognitive tests [106]. Future studies can incorporate these methodologies in validating social cognition tests. For instance, Bertoux et al. [107] used single-photon emission computed tomography (SPECT) to investigate the neural correlates of the mini Social Cognition and Emotional Assessment (SEA) [108] and assess its utility for evaluation and diagnosis in behavioural variant frontotemporal dementia. Such knowledge can inform models for clinical intervention and rehabilitation.

## 8. Conclusion

Social cognition tests can provide clinically useful information if used with other techniques as part of routine assessment protocols following cerebrovascular disease. The present review lists and describes some commonly used social cognition tests that can be incorporated into standard clinical evaluations. I have also highlighted some of the steps and considerations one should take in screening patients for social cognition deficits and suggested directions for future research aimed at improving the assessment value of social cognition tests. A key proposal in this review is that current evidence underscores the clinical importance of social cognition deficits in patients with neurological illness and that there is a crucial need to evaluate these deficits and prescribe informed rehabilitative interventions.

## Figures and Tables

**Table 1:** A list of some available social cognition tests.

Domain and tests	Description of test
*Theory of mind*	
False belief tasks	Participants have to understand that protagonists in the test hold false beliefs about the reality of a scenario.
Reading the Mind in the Eyes—revised [109]	Assesses the ability to decode expressed feelings and thoughts from pictures of eyes.
*Emotion recognition*	
Ekman 60 [110]	Assesses the ability to recognise 6 prototypical facial expressions of emotion (happiness, surprise, fear, sadness, disgust, and anger) from pictures of faces modelling these emotions.
Emotion Hexagon test [110]	Assesses recognition of facial expressions of the 6 basic emotions of happiness, surprise, fear, sadness, disgust, and anger from computer-generated images morphed between facial expressions to create different levels of recognition difficulty.
Profile of nonverbal sensitivity [111]	Measures the ability to decode affective interpersonal nonverbal cues from the face, body, and voice tone.
Florida Affect Battery [112]	Assesses capacities for the recognition of emotion from facial expressions and tone of voice.
Facial Emotion Identification Test (FEIT) [113]	Assesses participants' ability to identify and discriminate six emotions: happiness, sadness, anger, surprise, shame, and fear.
Bell-Lysaker Emotion Recognition Task (BLERT) [114]	Assesses the ability to recognise seven affective states from facial expressions, voice prosody, and upper-body movement cues.
*Social judgement*	
Social Awareness Test [115]	Assesses participants' judgements of the appropriateness of behaviour in social settings.
*Empathy*	
Balanced Emotional Empathy Scale [116]	Measures emotional empathy.
Interpersonal Reactivity Index [117]	The IRI [93] contains four subscales: (1) perspective taking, (2) fantasy, (3) empathic concern, and (4) personal distress. Pairs 1 and 2 provide a measure of cognitive empathy and pairs 3 and 4 give a measure of affective empathy.
The Empathy Quotient [118]	The Empathy Quotient (EQ) is a 60-item questionnaire (there is also a shorter, 40-item version) designed to measure empathy in adults. A children's version (EQ-C) derived from the (EQ) is also available.
Basic Empathy Scale [119]	The BES is a 20-item Likert-type scale that distinguishes between cognitive and affective empathy.
The Questionnaire Measure of Emotional Empathy (QMEE) [120]	The QMEE contains seven subscales and assesses an individual's tendency to react strongly to another's experience (there is also the Balanced Emotional Empathy Scale which is a follow-up of the QMEE and assesses emotional empathy).
Hogan Empathy Scale (HES) [121]	The HES is a 64-item instrument and has four separate dimensions: social self-confidence, even temperedness, sensitivity, and nonconformity.
*Broader tests*	
The Awareness of Social Inference Test (TASIT) [122]	Assesses emotion recognition, ToM reasoning, and the ability to make social inferences.
Faux Pas Recognition Test [123]	Measures ability to understand other people's mental states and also to empathise with them.
